# A Retinotopic Spiking Neural Network System for Accurate Recognition of Moving Objects Using NeuCube and Dynamic Vision Sensors

**DOI:** 10.3389/fncom.2018.00042

**Published:** 2018-06-12

**Authors:** Lukas Paulun, Anne Wendt, Nikola Kasabov

**Affiliations:** ^1^Knowledge Engineering and Discovery Research Institute, Auckland University of Technology, Auckland, New Zealand; ^2^Mathematical Institute, Albert Ludwigs University of Freiburg, Freiburg im Breisgau, Germany

**Keywords:** Spiking neural networks (SNN), NeuCube, dynamic vision sensor (DVS), MNIST-DVS, retinotopy, deep learning in SNN

## Abstract

This paper introduces a new system for dynamic visual recognition that combines bio-inspired hardware with a brain-like spiking neural network. The system is designed to take data from a dynamic vision sensor (DVS) that simulates the functioning of the human retina by producing an address event output (spike trains) based on the movement of objects. The system then convolutes the spike trains and feeds them into a brain-like spiking neural network, called NeuCube, which is organized in a three-dimensional manner, representing the organization of the primary visual cortex. Spatio-temporal patterns of the data are learned during a deep unsupervised learning stage, using spike-timing-dependent plasticity. In a second stage, supervised learning is performed to train the network for classification tasks. The convolution algorithm and the mapping into the network mimic the function of retinal ganglion cells and the retinotopic organization of the visual cortex. The NeuCube architecture can be used to visualize the deep connectivity inside the network before, during, and after training and thereby allows for a better understanding of the learning processes. The method was tested on the benchmark MNIST-DVS dataset and achieved a classification accuracy of 92.90%. The paper discusses advantages and limitations of the new method and concludes that it is worth exploring further on different datasets, aiming for advances in dynamic computer vision and multimodal systems that integrate visual, aural, tactile, and other kinds of information in a biologically plausible way.

## Introduction

During the past years, the quest for accurate image recognition systems has been one of the driving forces behind major advances in the field of artificial neural networks such as the development of convolutional neural networks (Lecun et al., 1998). Today, algorithms for image recognition are well advanced and can be found in many applications such as search engines, security systems, industrial robots, medical devices, and virtual reality. Besides the many areas of application, another reason for the fast progress in image recognition might be the vast knowledge about the human visual system. The eye is arguably the best studied human sensory organ and the visual cortex has been the main object of interest in a large number of neuroscientific studies. Findings from vision science have inspired the development of new hardware as well as novel algorithms and computational tools. High-definition and high-speed cameras have long surpassed the capacities of the human eye in terms of spatial and temporal resolution. On the software side though, it still proves to be a difficult task to extend the scope of present achievements in static image recognition to dynamic visual recognition of moving objects or a moving scene.

The benefit of accurate and fast dynamic visual recognition is apparent: each of the above-mentioned applications of image recognition constitutes a potential application area for dynamic visual recognition systems. Any kind of robot that must navigate within a three-dimensional environment or perform tasks on moving objects would benefit from an accurate and fast dynamic visual system. The popular topic of self-driving cars is only one example. Other potential implementations include security systems, automated traffic prediction and tolls, monitoring of manufacturing processes, navigational tools in air and ship traffic, or diagnostic assistants for inspections or surgery. Since the human visual system's adaptability and efficiency are still highly superior to computer systems when it comes to tasks of dynamic vision, it is natural to let biology serve as an inspiration for the development of new computational models.

Previous works have used a combination of bio-inspired visual sensors and spiking neural networks for the recognition of human postures (Perez-Carrasco et al., 2010), the extraction of car trajectories on a freeway (Bichler et al., 2012), or the control of robotic movements (Jimenez-Fernandez et al., 2009; Perez-Peña et al., 2013). We consider these very promising approaches, though the mentioned works lack benchmarking results that make them comparable.

This paper introduces a new system for dynamic visual recognition that combines a silicon retina device with a brain-like spiking neural network (SNN). As we introduce the different parts of our proposed system, we include findings from vision science that inspired us or that might provide promising approaches for future improvements. We present the setup and the results of a benchmarking experiment carried out on the MNIST-DVS dataset and show that our system achieves a classification accuracy of 92.90% on this dataset. The SNN architecture NeuCube is very flexible in terms of its connectivity and learning algorithms and allows for the visualization of the learning processes inside the SNN. After discussing the advantages and limitations of the system, we conclude by suggesting further exploration of the system's performance with modified algorithms and different datasets.

## The proposed system architecture

### The dynamic vision sensor

The Dynamic Vision Sensor (DVS) was developed at the Institute for Neuroinformatics in Zürich as a fast and storage efficient silicon retina system (Delbruck, 2008). Unlike conventional frame-based video cameras that capture multiple frames per second and store a large number of pixels for each of these frames, the DVS only captures changes in the brightness of single pixels caused by movement of the scene or an object (Lichtsteiner et al., 2008). This is called an Address Event Representation (AER) since the output of the sensor consists of a time series of events together with their location (address), representing the temporal contrast of a specific pixel at a specific time. By responding to temporal contrast on the pixel-level rather than taking a continuous series of snapshots of the whole scene, the DVS mimics the functioning of the human retina much better than conventional video cameras (Purves, 2012).

Together with its focus on movements within a scene there is another reason to choose the DVS over a conventional video camera for a dynamic vision system based on a spiking neural network: the address event output of the DVS comes in the form of a series of spike trains, each spike train corresponding to one pixel of the sensor. Every single spike in the train of one specific pixel represents a change in brightness in that pixel at a specific time. However, there are two difficulties with taking the raw DVS output as spike trains and directly feeding them into a spiking neural network: firstly, the sensor can achieve a very high temporal resolution of 1 μs and a spike train for a single pixel will initially consist of many time steps, e.g., 2,000,000 time steps for a 2 s video, and a relatively small number of spikes. Feeding such a spike train into a spiking neural network would result in very low overall spiking activity and probably unsatisfying performance. Secondly, although the sensor's spatial resolution of 128 × 128 = 16,384 pixels is low compared to conventional video cameras, it is desirable to reduce computational cost by integrating the signals of multiple pixels into single input neurons for the SNN rather than creating 16,384 input neurons.

For this purpose, we propose an algorithm for the compression of time and the convolution and pooling of the DVS pixels into a total of 128 spike trains consisting of roughly 100 time steps for each second of video data that can then be fed into 128 input neurons of an SNN.

### Proposed encoding algorithm of DVS data as input data for the SNN system

The algorithm we propose is inspired by the structure and organization of retinal ganglion cells. These cells receive information from photoreceptors on the retina and transmit them to the brain (Purves, 2012). There are different types of retinal ganglion cells, but we focus on two global properties shared by the majority of all ganglion cells: first, the distribution of retinal ganglion cells across the retina, which is used to determine which photoreceptors converge into one retinal ganglion cell and, thus, how many DVS pixels converge into one input neuron for our SNN. Second, the mechanism by which retinal ganglion cells fire and, thus, the algorithm that generates the input spike trains for the SNN.

#### Pooling of DVS output into 128 input neurons of the SNN system

Despite large differences across individuals, there are roughly 100 million photoreceptor cells on the retina and around 1 million retinal ganglion cells providing information transmission to the brain (Curcio et al., 1990). Thus, on average, one ganglion cell integrates information from roughly 100 photoreceptor cells. However, the number of photoreceptors converging into one ganglion cell depends highly on the retinal location of the photoreceptors. Ganglion cells connecting to the *fovea centralis*, the small central spot of the retina specialized in sharp and detailed vision, receive information from only a single photoreceptor cell, implying that information from these photoreceptors is transmitted directly to the brain without any pooling (Purves, 2012). The receptive fields of ganglion cells increase with distance from the fovea and ganglion cells connecting to peripheral parts of the retina integrate the signals of many photoreceptors at once (Croner and Kaplan, 1995).

**Figure 1:**
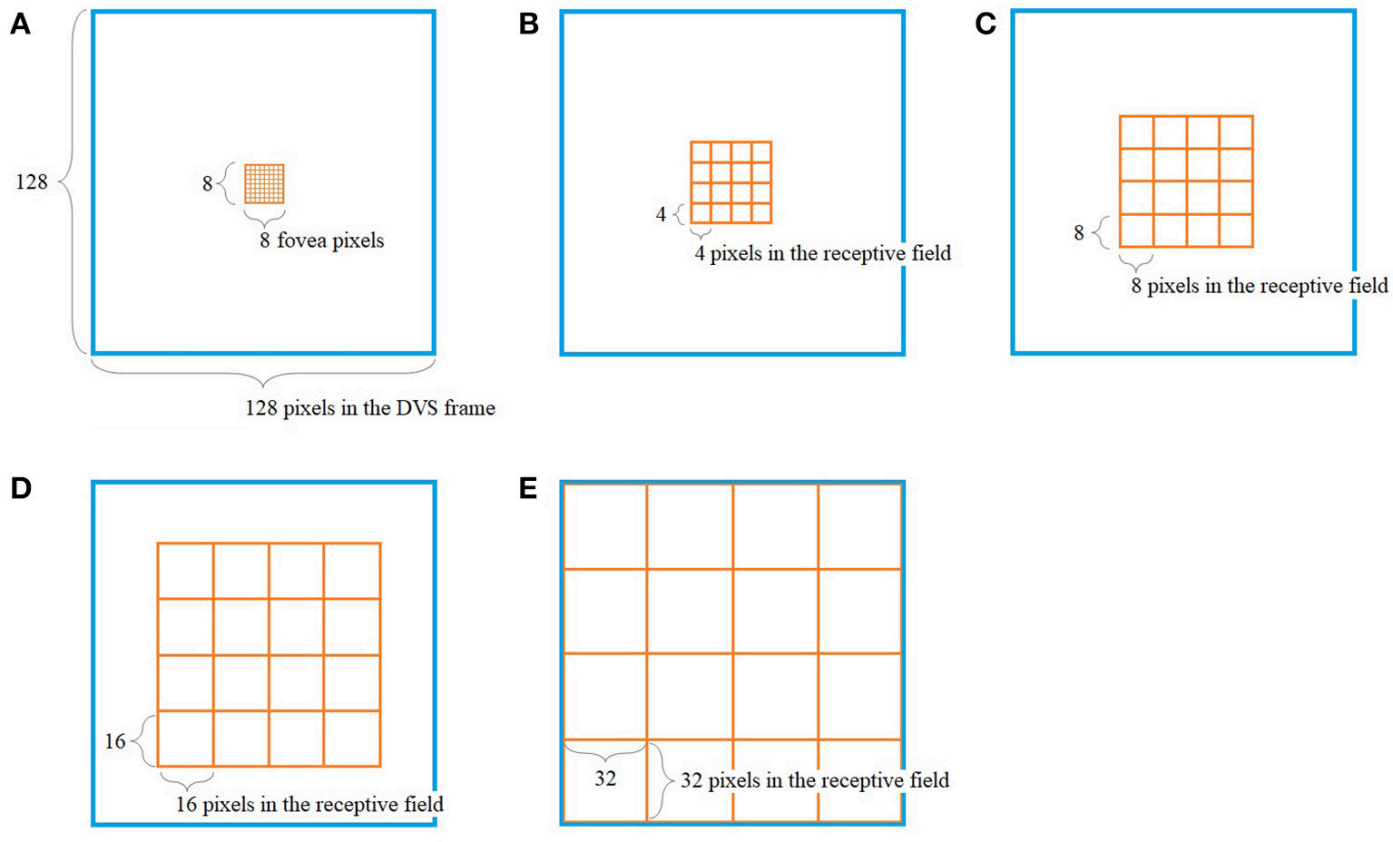
Pooling of 128 × 128 DVS pixels into 128 ganglion cells. 64 foveal ganglion cells that correspond to the central 64 DVS pixels **(A)** and four groups of 16 ganglion cells each with increasing size of receptive fields toward the periphery **(B–E)**. The image seen by the DVS camera is marked with a blue frame and the receptive fields are marked with orange frames.

The way our encoding algorithm pools information from multiple DVS pixels into single spike trains adapts this property of detailed information transmission from central parts of the retina and averaging over larger numbers of photoreceptors in the periphery. Overall, the algorithm generates 128 spike trains that will serve as input for the SNN. Each spike train represents one retinal ganglion cell with its own receptive field on the 128 × 128-pixel output of the DVS (Figure 1).

In our algorithm, the central 8 × 8 pixels of the DVS output represent the fovea (Figure 1A), and for each of these central 64 pixels, there is a single ganglion cell only considering the output of that single pixel. Furthermore, there are four groups of 16 ganglion cells each, with receptive fields that increase from the center to the periphery. The first group consists of the central 16 × 16 pixels, divided into 16 squares that integrate an area of four by four pixels each (Figure 1B). The next group consists of the central 32 × 32 pixels, again divided into 16 squares, this time with an area of 8 × 8 pixels each (Figure 1C). The same happens for the central 64 × 64 pixels (Figure 1D) and the total of 128 × 128 pixels (Figure 1E), resulting in 16 squares per group, of size 16 × 16 and 32 × 32, respectively. In this pooling mechanism, an average of 170.5 pixels converge into one ganglion cell. The size of the receptive fields can easily be adapted to higher or non-square video resolutions.

Having set the distribution of the ganglion cells across the DVS output, the next step is to determine how the information of the DVS pixels is encoded into spike trains for the ganglion cells.

#### Firing mechanism

The Dynamic Vision Sensor provides a very high temporal resolution of up to 1 μs. Preserving is detailed temporal information is desirable from a computational point of view, but as described below we reduce this resolution to 10 ms to maintain biological plausibility. While some spike encoding algorithms like Poisson models focus merely on the spike count within a given time interval and disregard the exact spike timing, it has been shown that the spike timing of mammalian retinal ganglion cells conveys several times more information than the spike count (Berry et al., 1997; van Rullen and Thorpe, 2001; Uzzell and Chichilnisky, 2004). Furthermore, retinal ganglion cells fire very briefly as a response to specific stimuli rather than emitting a high frequency of background firing. Spikes emitted by retinal ganglion cells of rabbits and salamanders, presented with random flicker, covered less than 5% of the total stimulus time (Berry et al., 1997). The maximum firing rate of retinal ganglion cells varies between different animal species and depends on the type of visual stimuli. Transient peak rates of up to 250 Hz have been observed in retinal ganglion cells of mice (Krieger et al., 2017), but for the sustained firing of human retinal ganglion cells, an upper bound of 100 Hz can be reasonably assumed (Nelson, 1995).

As described in section The Dynamic Vision Sensor, the DVS output consists of a series of events, including their timing in microseconds and their location in pixel coordinates. In fact, each event also includes a polarity of +1 or −1, depending on whether the event indicates a pixel becoming brighter or darker. Our encoding algorithm ignores the event polarity, but it might be worthwhile for future experiments to consider a translation of positive and negative events into positive and negative spikes.

**Figure 2:**
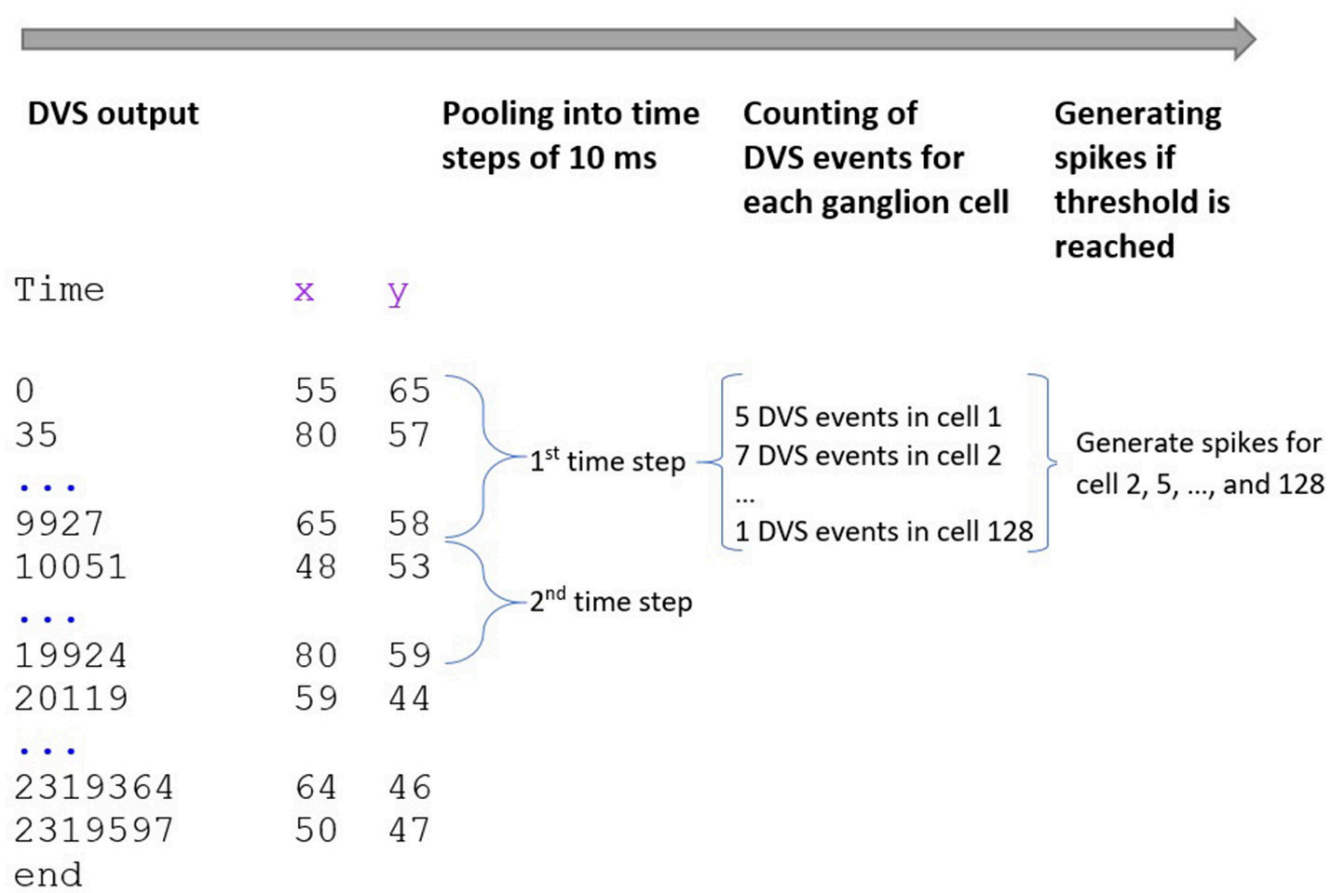
Encoding of spike trains from DVS output. The DVS time series is grouped into windows of 10 ms. For each time step, the DVS events within the receptive fields of all 128 ganglion cells are counted. If the number of DVS events within the receptive field of one ganglion cell exceeds a certain threshold, the cell fires at that time step.

Our spike encoding algorithm is illustrated in Figure 2. In the first step, the algorithm takes the time series of the DVS and groups it into windows of 10,000 μs or 10 ms. The new time series consists of 10 ms steps, and for every ganglion cell, it must be decided at which of these steps the cell will fire. Since each time step represents 10 ms of video data, the maximum firing rate of the ganglion cells cannot exceed 100 Hz. The encoding for the central 64 pixels that represent the fovea is straightforward: if there is at least one event for a pixel at time step t_i_, the ganglion cell that corresponds to that pixel will fire at t_i_. There are no parameters to tune for these central 64 pixels and the spike trains of the ganglion cells that correspond to these pixels are completely determined by the DVS output. For the 64 ganglion cells that integrate the events of multiple DVS pixels, the situation is slightly different. For each of these cells, the algorithm counts how many events occurred in each time window within the receptive field of that ganglion cell. If the number of events from pixels within the receptive field of cell C_j_ at time step t_i_ exceeds a certain threshold, C_j_ will fire at t_i_.

Theoretically, this threshold can be set for each ganglion cell individually, but since the 16 cells of each group have receptive fields of the same size, our algorithm assigns the same threshold to all 16 cells of a group, resulting in a total of 4 thresholds that can be tuned. Clearly, the value of the thresholds will determine the average spike rate of the final spike trains, with higher thresholds leading to fewer spikes, and it is possible to imitate biological evidence about spike rates under certain stimuli. We discuss the tuning of the thresholds in more detail in section Model Design and Implementation.

Inspired by the structure and organization of retinal ganglion cells, our algorithm pools 128 × 128 DVS pixels into 128 ganglion cells that will serve as input neurons for the SNN. The algorithm compresses the microsecond resolution of the DVS output into time steps of 10 ms, but it preserves the timing of the DVS events instead of generating a Poisson process with random spike timing. The next section describes the structure of a brain-like SNN architecture called NeuCube, and our imitation of the retinotopic mapping of retinal ganglion cells into the visual cortex.

### The brain-like SNN neucube and the proposed retinotopic mapping

**Figure 3:**
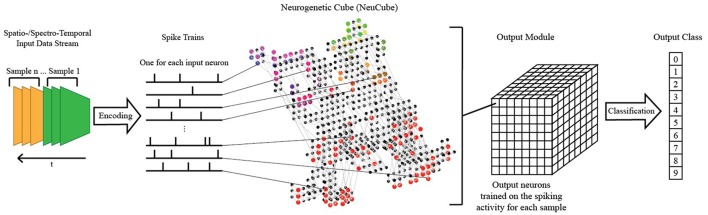
A schematic diagram of a general NeuCube architecture, consisting of: input encoding module; NeuCube module; output function module. Our system only makes use of the NeuCube module and the output function module.

**Figure 4:**
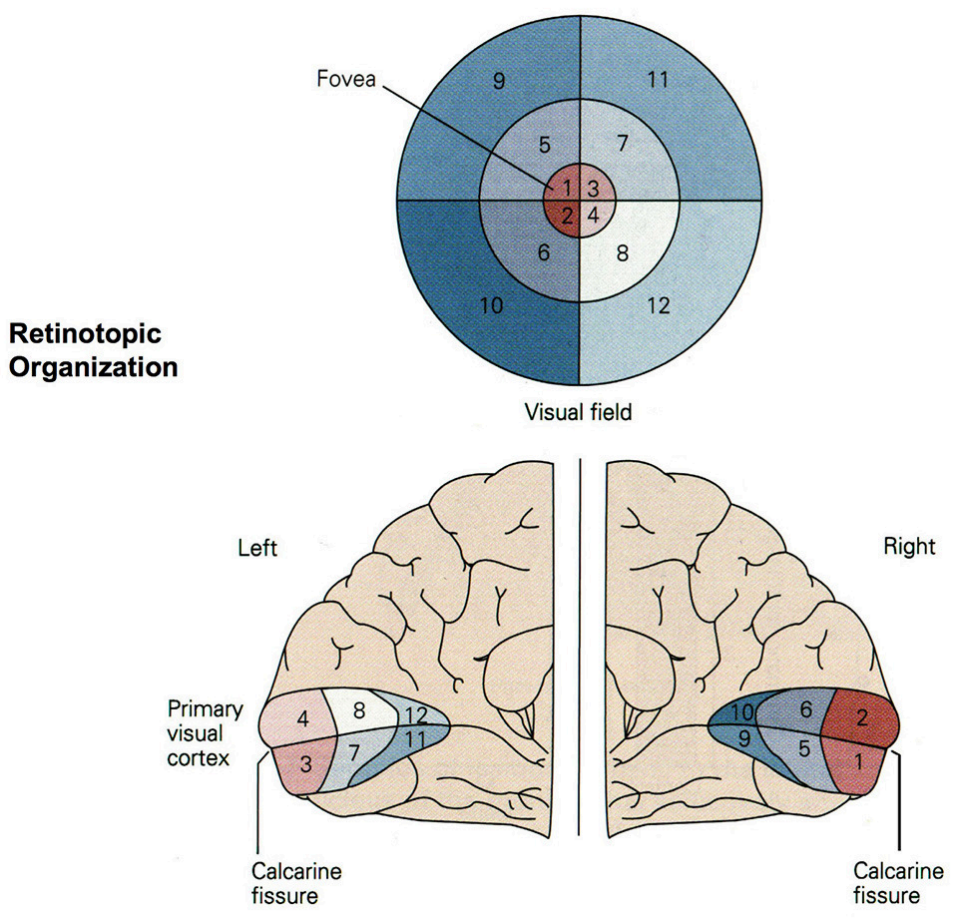
Retinotopic organization of the primary visual cortex. Up to 50% of the primary visual cortex processes foveal signals (cortical magnification). Signals from the top left of the visual field are mapped to the bottom right of the visual cortex (retinotopy). Source: Jaygandhi786 (2015).

The NeuCube SNN architecture incorporates several different principles of SNN and combines them into a single model for mapping, learning, and understanding of spatio-temporal data (Kasabov, 2014). Signals are processed along successive stages as shown in Figure 3. Before going into detail about the learning algorithms used by NeuCube, we want to focus on the three-dimensional structure of NeuCube and the bio-inspired way we mapped the 128 input neurons into this structure. Our system uses a NeuCube initialized with 732 neurons, using the MNI coordinates of neurons from the primary visual cortex (V1, Brodman area 17), taken from the Atlas of the Human Brain (downloaded together with the xjView toolbox: http://www.alivelearn.net/xjview). The number of neurons is only bounded by computational limitations; it is possible to add further neurons from the secondary or tertiary visual cortex or to represent the whole brain. Initial connections between the neurons are based on the “small-world” paradigm, where random connections are formed within a pre-defined maximum distance of each neuron, 80% of the time as excitatory and 20% of the time as inhibitory connections. The mapping of the 128 input neurons into the 732 neurons of NeuCube mimics two important characteristics of the human visual cortex: cortical magnification and retinotopic mapping (Figure 4).

Cortical magnification describes the overrepresentation of foveal signals inside the primary visual cortex. Although the fovea has a diameter of only 1.2 mm (Purves, 2012), its signals are processed by almost 50% of all neurons in V1 (Krantz, 2012; Born et al., 2015). Therefore, we chose exactly 64 of our 128 input neurons to correspond to the central 64 DVS pixels with a one-to-one relationship. This way, 50% of input neurons automatically correspond to the central pixels of the DVS, just like 50% of the primary visual cortex correspond to the central photoreceptors on the retina.

**Figure 5:**
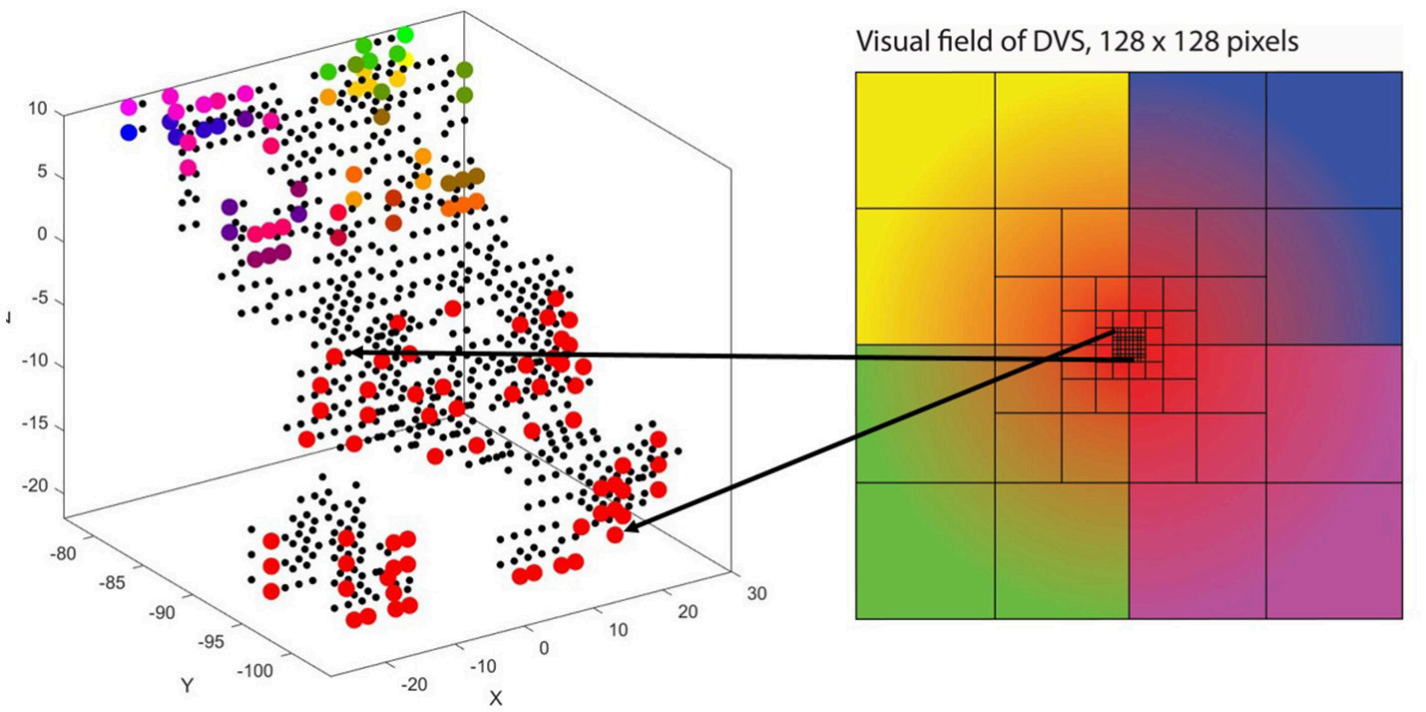
Retinotopic mapping of 128 input neurons to NeuCube, initialized with 732 neurons, using the MNI coordinates of neurons from the primary visual cortex, taken from the Atlas of the Human Brain.

The second characteristic of the primary visual cortex that we adopted in our mapping is the preservation of spatial relationships between photoreceptors on the retina and their neural representation in the primary visual cortex, the so-called retinotopy (Rosa, 2002). Signals from the top left of our visual field are mapped to the bottom right of V1 and vice versa. What humans see is flipped upside down and mirrored, but objects that appear next to each other in the visual field will still be represented next to each other in V1. Both the foveal as well as the peripheral ganglion cells follow this principle, although foveal signals are mapped into the posterior part and peripheral signals into the anterior part of V1 (Purves, 2012). Figure 5 shows how the principle of retinotopy is applied to the mapping of the 128 input neurons to the 732 neurons of NeuCube.

### Unsupervised and supervised learning of dynamic visual patterns in the neucube architecture

Learning in the NeuCube is performed in two stages: in the first step, unsupervised learning is performed to modify the initial connection weights. In our system we use pair-based multiplicative spike-timing-dependent plasticity (STDP, van Rossum et al., 2000), but in principle, the NeuCube architecture allows for a flexible implementation of different learning algorithms. The SNN will learn to activate the same groups of spiking neurons when similar input stimuli are presented and to change existing connections that preserve the spatio-temporal patterns of the input data (Kasabov and Capecci, 2015). Previous works have shown that STDP is well suited to train neurons to respond to discriminative visual features (Masquelier and Thorpe, 2007). The neurons become selective to successive coincidences of particular patterns and learn to detect them robustly even in the presence of noise (Masquelier et al., 2009). Our approach using the NeuCube differs from these works mainly in the structure of the network, which is not based on layers, but rather a three-dimensional network shaped like the primary visual cortex. However, our results are similar to those works in that certain neurons and connections can be identified that seem to play a major role in discriminating between the different classes. NeuCube allows for a visualization of the learning process and we discuss how the visualization can be used for a better understanding of the data and the neural processes after presenting our experimental results.

In the second step, supervised learning is applied to the spiking neurons in the output classification module, where the same spike trains used for the unsupervised training are now propagated again through the trained SNN and output neurons are generated and trained to classify the spiking activity of the SNN into pre-defined classes (Kasabov and Capecci, 2015). Again, the NeuCube architecture allows for the application of different algorithms for the evolving classifier. The output function we used is called the dynamic evolving SNN algorithm (deSNN, Kasabov et al., 2013), which makes use of rank-order learning (Thorpe and Gautrais, 1999). This kind of evolving classifier is computationally inexpensive and puts emphasis on the order in which input spikes arrive, making it suitable for on-line learning and early prediction of temporal events (Kasabov, 2014). Similar to previous works on image recognition based on reward-modulated STDP (Mozafari et al., 2017), the deSNN algorithm uses a “highest” layer of neurons to discriminate between classes. While Mozafari et al. (2017) used an existing layer of output neurons, the deSNN algorithm creates and trains one new output neuron per sample by connecting it to all 732 neurons in the network and propagating the signal through the network once more. The connection weights that are learned in this process are then classified using a K-nearest neighbor (KNN) algorithm and the labels that are known for all the samples. Here our method differs from the aforementioned (Mozafari et al., 2017) in that we do not apply “anti-STDP” for misclassified samples before applying KNN. This means that the results of the deSNN's decisions are not fed back into the network since we create a new output neuron for each sample.

For a more detailed description of the NeuCube architecture see Kasabov (2014).

### Summary of the proposed methodology

The methodology we propose for dynamic visual recognition consists of the following steps:

1. Event-based video recording with DVS.
2. Pooling and encoding of DVS output into spike trains for the input neurons of the SNN.
3. Training NeuCube on the spike data using unsupervised learning, e.g., STDP.
4. Training of an output classifier in a supervised mode.
5. Validating the classification results.
6. Repeating steps (2–5) for different parameter values to optimize the classification performance. Recording the model with the best performance.
7. Visualizing the trained SNN and analyzing its connectivity and spiking activity for a better understanding of the data and the involved brain processes.

We present the application of this method on a benchmarking experiment with the MNIST-DVS dataset for spike-based dynamic visual recognition and go into further detail about the tuning of parameters and analysis of the SNN.

## Benchmarking on the MNIST-DVS dataset

### Description of the MNIST-DVS dataset

The MNIST dataset of handwritten digits (Lecun et al., 1998) has been one of the most popular benchmarking datasets for image recognition for over 20 years. With the advent of spiking neural networks, MNIST has naturally been used as a benchmark for spike-based visual recognition systems (Brader et al., 2007; Querlioz et al., 2013; Diehl and Cook, 2015; Zhao et al., 2015; Kheradpisheh et al., 2017). However, these works only account for the recognition of the static MNIST pictures and do not aim toward dynamic visual recognition of moving objects. An important part of the functioning of spiking neural networks is the dimension of time within the spike trains and on datasets that also have such a temporal dimension, spiking neural networks might be superior to classical artificial neural networks.

The NE15-MNIST database (Neuromorphic Engineering 2015 on MNIST, Serrano-Gotarredona and Linares-Barranco, 2015; Liu et al., 2016) that we used for our study is based on the original MNIST dataset. NE15-MNIST consists of four subsets that all aim to provide a benchmark for spike-based visual recognition. While the *Poissonian* and the *FoCal* subsets are synthetically generated from static MNIST images, the other two subsets are based on 128 × 128 pixel DVS recordings of the MNIST images. The MNIST-FLASH-DVS subset contains DVS recordings of MNIST digits that are flashed on a screen. Because we were interested in dynamic visual recognition of moving objects, we decided to work on the MNIST-DVS subset that consists of DVS recordings of MNIST digits that move back and forth across a screen and thereby produce temporal contrast and DVS events on the digits' edges.

The MNIST-DVS dataset is available online (Yousefzadeh et al., 2015). It consists of 30,000 recordings of 10,000 original MNIST digits recorded at three different scales each (scale-4, scale-8, and scale-16). Each recording has a time length of about 2.5 s, during which the digit moves twice from a position at the bottom left of the middle of the screen to the top right and back. The files are provided in the jAER format (Delbruck, 2008) and the dataset includes Matlab scripts for a conversion to Matlab arrays and three kinds of data preprocessing: removal of a 75 Hz timestamp harmonic produced by the LCD screen, stabilization of the digits on the center of the screen and removal of the event polarity information.

Previous classification results on the MNIST-DVS dataset are shown in Table 1. Henderson et al. (2015) derive a new event-based learning scheme and apply it to a layered feedforward spiking neural network, which is trained self-supervised for classification of the MNIST-DVS digits. Zhao et al. (2015) use a composite system, consisting of a convolutional spiking neural network for feature extraction and a network of tempotron neurons for spike-based classification. While these two systems are fully event-driven, Stromatias et al. (2017) use a combination of a spiking neural network and a conventional artificial neural network. A convolutional SNN is used to capture the temporal dynamics of the DVS data and create a new, frame-based dataset, which is fed into a fully-connected artificial neural network. The supervised learning itself then takes place in this non-spiking network, using a stochastic gradient descent algorithm. In our concluding remarks we suggest how this approach could be combined with our model to maintain the high classification accuracies while providing greater biological plausibility.

**Table 1:** Previous classification results on the MNIST-DVS dataset.

	**Network type**	**Learning algorithm**	**Total number of samples used**	**Train-test-ratio**	**Classification on test set (%)**
Henderson et al., 2015	Feedforward SNN	A new scheme for spike-based learning	10.000 (scale not mentioned)	90–10	87.41
Zhao et al., 2015	Composite system, including convolution, motion detector, feature spike conversion, and SNN classifier	Tempotron learning	10.000 (scale-4)	90–10	88.14
Stromatias et al., 2017	Composite system, including convolutional SNN, non-spiking fully connected classifier, and spiking output layer	Stochastic Gradient Descent (inside the non-spiking classifier)	10.000 (scale-16)	80–20	97.95

### Model design and implementation

The only preprocessing we applied to the data was the removal of the 75 Hz timestamp harmonic. Stabilizing the video data would have been contrary to our intention to develop a system for dynamic visual recognition, and in fact, preliminary experiments suggested that the system would perform better on the original unstabilized videos. To run our spike encoding algorithm on the data, we used the script provided with the dataset to convert the jAER files into Matlab arrays.

The pooling of the DVS spikes into 128 input spike trains (ganglion cells) for the SNN, as described within section The Proposed System Architecture, remained the same throughout all experiments. Inside the spike encoding algorithm, only those four thresholds were changed that determine how many pixels within the receptive field of a ganglion cell must fire within one time step to make the ganglion cell itself emit a spike. As a first step, we wanted to find out how the system would perform differently when these thresholds and, thus, the average spike rate of the input data for the SNN, were changed. As described in section Firing Mechanism, the ganglion cells' receptive fields decrease from the periphery toward the center. Starting from the periphery, ganglion cells in group 1 integrate the signal of 32 × 32 = 1.024 DVS pixels, cells in group 2 from 16 × 16 = 256 pixels, cells in group 3 from 8 × 8 = 64 pixels, and cells in group 4 from 4 × 4 = 16 pixels. Assigning the same percentage threshold to all four groups would result in very low or no activity in the peripheral ganglion cells, e.g., with a threshold of 10% it would take only two DVS events within the receptive field of a ganglion cell in group 4 to trigger a spike, but 103 DVS events within the receptive field of a ganglion cell in group 1. Especially with the MNIST-DVS dataset, where DVS events only occur at the edges of the moving digits and not in larger blobs, this would make the peripheral ganglion cells redundant. On the other hand, increasing the thresholds too much from group to group toward the center would put more emphasis on the peripheral parts of the video than intended.

**Figure 6:**
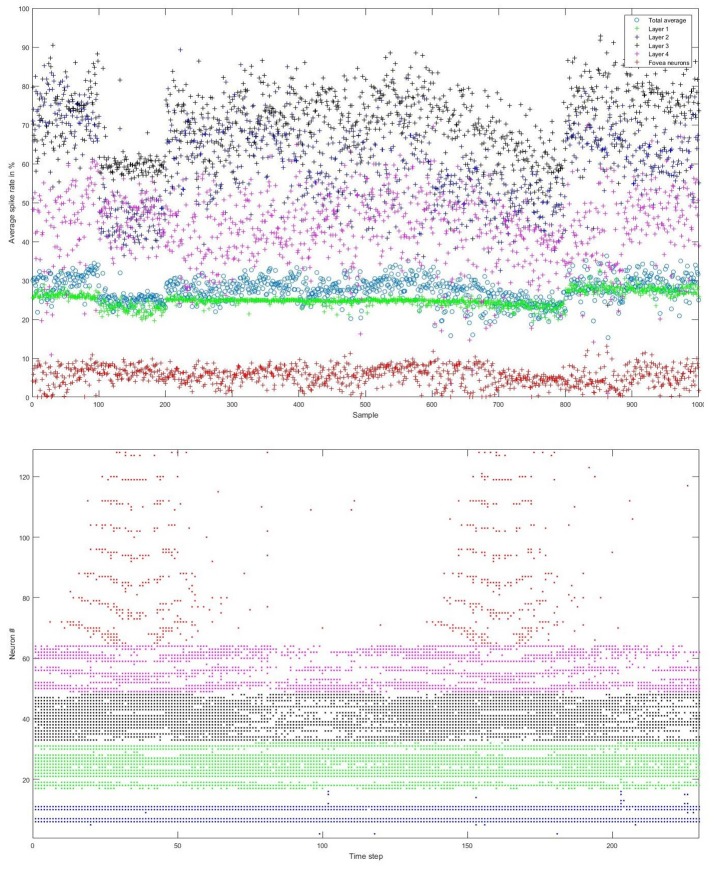
**Top**: Average spike rates of 1,000 scale-8 videos (100 per digit) resulting from encoding-thresholds of 0.5, 1, 2, and 4% for the four groups of retinal ganglion cells, respectively (from periphery to center). Average spike rates are measured in %, dividing the average number of time steps in which the cells of a given group fired by the total number of time steps. The total average spike rate is 27.57%. **Bottom**: Example of encoded spike trains for one sample (digit 0, scale-8, sample #1). Neurons 1–16, 17–32, 33–48, and 49–64 represent the four groups of ganglion cells from the periphery to the center; neurons 65–128 represent the foveal ganglion cells. The spike pattern of the foveal ganglion cells clearly represents the two times that the digit moves across the center of the screen.

We carefully watched the MNIST-DVS videos and compared the distribution of DVS events with the average spike rates for the groups of ganglion cells that were produced by different spiking thresholds. We found that increasing the percentage thresholds by a factor of two from group to group toward the center would preserve the distribution of DVS events relatively well and not put too much emphasis on any single group. Figure 6 shows the average spike rates for 1,000 scale-8 videos (100 per digit), produced by thresholds of 0.5% for group 1, 1% for group 2, 2% for group 3 and 4% for group 4. Since time is discrete in our model, we measure the average spike rates in %, dividing the number of time steps in which a cell fired by the total number of time steps. Most spikes occur in groups 2 and 3, consistent with the general distribution of DVS events in the scale-8 videos. The total spike average of the samples shown in Figure 6 is 27.57%. We altered the thresholds to get clearly distinguishable total spike averages. Table 2 shows four different choices of thresholds, resulting in average spike rates of roughly 7, 14, 26, and 32% (exact numbers vary between different video scales). The last row represents the maximal achievable average spike rate with a threshold of 0% for each group. In that case, every ganglion cell fires if there is at least one DVS event in its receptive field at a given time step.

**Table 2:** Different choices of spike thresholds within the spike encoding algorithm and corresponding average spike rates.

**Spike threshold in %**				**Approximate average spike rate (%)**
**Group 1**	**Group 2**	**Group 3**	**Group 4**	
5	10	20	40	5
2.5	5	10	20	13
0.5	1	2	4	26
0	0	0	0	32

The mapping of the input spikes into the SNN NeuCube was done according to the proposed retinotopic mapping and it remained the same throughout all experiments. In all experiments NeuCube was initialized with 732 leaky integrate and fire neurons (LIF), representing the primary visual cortex. For future experiments with higher video resolutions and more input neurons, NeuCube can easily be extended to include neurons that represent the secondary and the tertiary visual cortex. Initial connections are formed following “small-world” connectivity with random connections within a predefined maximum distance from each neuron. This maximum distance was set to 2.5 in all experiments.

As described previously, unsupervised learning using STDP is performed first to learn spatio-temporal patterns by forming new connections between neurons, before the output classifier is trained in a supervised manner using the dynamic evolving SNN (deSNN) algorithm (Kasabov et al., 2013). The NeuCube architecture is a stochastic model and, therefore, sensitive to parameter settings. To find the best values for the major parameters that influence the system's performance, we applied a grid search method that tests the system on different combinations of parameters within a predefined range and used those parameter values that resulted in the best classification accuracy. For the firing threshold, the refractory time and the potential leak rate of the LIF neurons we used values of 0.5, 6, and 0.002, respectively. The STDP learning parameter was set to 0.01. The variables *Mod* and *Drift* of the deSNN classifier were set to 0.8 and 0.005. See Kasabov and Capecci (2015) for a more detailed explanation of these parameters.

### Experimental results

To compare the system's performance, we performed 10-fold cross-validation on 1,000 videos (first 100 of each digit), with 900 videos used for training and 100 for testing in each fold, for different video scales and average spike rates. Table 3 summarizes the results. As a general trend, with few exceptions, the classification accuracy increased together with the average spike rate of the input neurons. For all video scales, the classification accuracy also increased when the system was run on all 10,000 videos of a given scale. The best classification results were achieved with all 10,000 videos of one scale, encoded with the highest possible spike rate (0% as spike encoding threshold for all four groups). Classification accuracies were 90.56, 92.03, and 86.09% % for scale-4, scale-8, and scale-16, respectively. The best accuracy in a single run with 90% of randomly selected data samples for training and the remaining 10% for testing was 92.90% for 10,000 scale-8 videos with the highest possible spike rate. This result is comparable to previous results on the MNIST-DVS dataset, presented in Table 1.

**Table 3:** Results of 10-fold cross validation for different video scales and average spike rates.

**Video scale**	**Number of samples**	**Average spike rate (%)**	**Classification accuracy (%)**
Scale-4	1,000	7.85	63.80
“	1,000	13.94	77.10
“	1,000	25.77	75.50
“	1,000	31.77	83.40
“	10,000	31.98	90.56
Scale-8	1,000	5.29	66.40
“	1,000	13.49	83.00
“	1,000	27.57	84.20
“	1,000	32.96	86.20
“	10,000	32.93	92.03
Scale-16	1,000	3.81	60.50
“	1,000	12.64	82.90
“	1,000	26.94	78.60
“	1,000	31.72	77.50
“	10,000	31.79	86.09

The lower accuracies on the scale-4 and the scale-16 samples reflect the fact that in these videos, the MNIST digits fill out either the whole screen (scale-16) or only a very little region in the center (scale-4). For the scale-4 digits, the signals transmitted by ganglion cells from groups 1, 2, and 3 are mostly noise and do not contain much information about the digits. In the scale-16 videos, there is almost no activity in the central region of the screen and, thus, no information is transmitted by the 64 foveal ganglion cells. Since our method puts heavy emphasis on the center of the video (50% of the input neurons represent data from only the central 64 pixels), performance on the scale-16 videos is lower.

### Model interpretation for a better understanding of the processes inside the visual cortex

**Figure 7:**
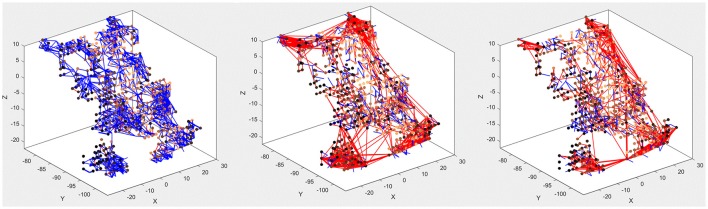
Connectivity of the SNN before **(left)** and after training on 1,000 scale-4 samples **(middle)** and 1,000 scale-16 samples **(right)**. During training, new connections are created while others vanish, representing relations between spiking neurons that evolve as a response to the spatio-temporal patterns of the data.

The main purpose of the above experiments, carried out on the MNIST-DVS dataset, is to confirm the system's classification performance on a benchmark dataset, and the moving digits do not represent a real-life scene. However, we want to show how the SNN can be analyzed after being trained, to see how its connectivity changes in response to the data. Figure 7 compares the connectivity of the SNN before and after unsupervised training on 1,000 scale-4 videos with the highest possible spike rate. Blue and red lines represent positive and negative connections, respectively. We can notice that some of the randomly created initial connections disappear during the training process. Instead, many new negative connections are created, mostly between neurons in the region that represents the posterior part of the primary visual cortex, where signals from the foveal ganglion cells arrive. Some of the new connections connect neurons over a long distance, especially in the very posterior part of the SNN, where a gap between neurons prevents the initial formation of “small-world” connections. As can be seen in Figure 5, the neurons on both sides of this gap represent adjacent DVS pixels, and by bridging this gap, the new connections allow for communication between these neurons. A comparison with the connectivity after training the SNN on 1,000 scale-16 videos shows that slightly fewer connections are formed between neurons processing foveal information since the scale-16 videos contain less DVS events in the foveal region. This effect is due to the acquisition hardware used and could be compensated for by the simulation of saccadic eye movements inside the encoding algorithm. In a biological retina, these rapid eye movements ensure that the fovea centralis focuses on salient features instead of constantly covering a less important area of the visual field. We discuss this possible improvement of the encoding algorithm in the next section.

**Figure 8:**
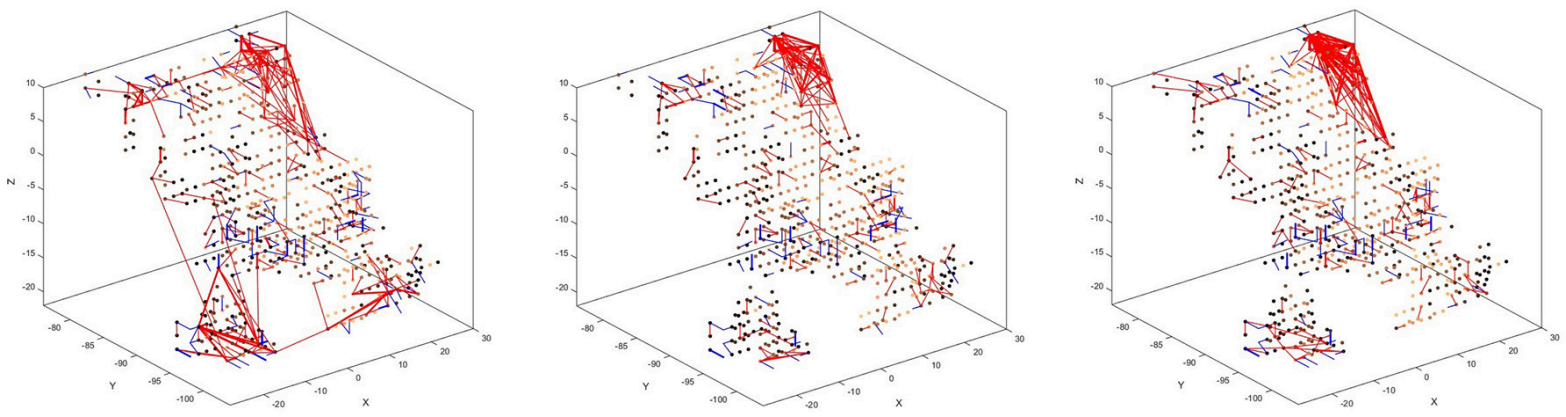
Connectivity of the SNN after unsupervised training on 1,000 scale-8 samples each for the three digits 1 **(left)**, 5 **(center)**, and 8 **(right)**. There is a visible difference between the connections, corresponding to the visual characteristics of the digits.

There is also a visible difference between connections created for different digits. Figure 8 shows the status of the network after unsupervised training using only digits 1, 5, and 8, respectively. Interestingly, the connections created for digits 5 and 8 look similar, just like the digits themselves have a similar shape. The connections created after training on digit 1, on the other hand, look distinctly different. We can, therefore, conclude that the visual characteristics of the digits are preserved in our system, just like they are in the human visual cortex.

## Discussion of the system's advantages and limitations

The proposed system achieves a classification performance on the benchmark MNIST-DVS dataset that can keep up with previous works on this dataset and is superior to those works that used a spiking neural network classifier. Every part of the system, the DVS sensor, the algorithm for encoding the DVS output into spike trains, and the SNN NeuCube adopt features from the human visual system. This allows for future experiments where the same stimuli are presented to humans and the proposed system and brain processes visualized by neuroimaging methods can be compared to the network processes of the SNN, which can be easily visualized within the NeuCube architecture.

Another advantage of the proposed system is the high flexibility of the SNN's three-dimensional structure. The NeuCube architecture is not restricted to consist of neurons that represent only the visual cortex. For example, one could map aural stimuli to input neurons representing the auditory cortex, to obtain a model that processes aural and visual information at the same time in a brain-like way. The integration of other kinds of data, such as tactile or olfactory information, within a multimodal model is conceivable as well.

We found that the system's classification performance increases together with the average spike rates of the 128 input neurons. To account for the findings of Berry et al. (1997) in retinal ganglion cells of rabbits and salamanders, we started our experiments with low spike rates of approximately 5%, but the classification accuracies were very low in these cases. However, the reported firing rates of rabbit and salamander ganglion cells were measured during the presentation of random flicker, which might yield very different firing behavior than stimuli like the moving digits. Single cell recordings of retinal ganglion cells could provide more evidence about the firing rates under specific stimuli. The parameters of the spike encoding algorithm that determine the average spike rates can then easily be tuned to mimic the behavior of real retinal ganglion cells and it would be interesting to see if classification accuracy increases when the average spike rates conform to the biological evidence.

Since so much is known about the human visual system and we aimed to develop a biologically plausible, yet computationally feasible implementation, there are many details not included in our model. There already exist very advanced mathematical models for the function of retinal ganglion cells (Wei and Ren, 2013) and our spike encoding algorithm has by far not touched every detail of them. The receptive field of each ganglion cell, for example, is split into a center region and a surrounding region with opposite behavior toward light (Nelson, 1995). In so-called on-center cells, the center region is stimulated, whereas the surrounding region is inhibited when exposed to light. So-called off-center cells exhibit converse behavior. Including the function of on- and off-center ganglion cells inside the spike encoding algorithm would highly increase the model's biological plausibility, but also its computational complexity. Another computational restriction of our model is that the random initial creation of excitatory and inhibitory connections causes a violation of Dale's Principle, which states that all axonal branches of a neuron perform the same chemical reaction.

One shortcoming of the DVS when compared to the human retina is its inability to process colors. The DVS only encodes temporal changes in brightness that signal motion (Delbruck, 2008), similar to the rod photoreceptors on the retina and the functionality of the magnocellular fibers in the optical nerve (Purves, 2012). However, the cone photoreceptors on the retina as well as the comparatively large amount of parvocellular fibers in the optic nerve are not modeled by the DVS despite their importance for detecting and transmitting information about color and details of the perceived objects (Purves, 2012). This means that all object recognition approaches using DVS input are somewhat limited because the DVS only captures signals that the human visual system would use to detect motion and distances to objects, but not those signals necessary for recognizing objects and details.

The proposed system puts strong emphasis on the central part of the videos in both the encoding of DVS events to spike trains and the representation inside the SNN. This is justified by analogous features of the fovea centralis in the center of the human retina, responsible for focused vision. However, there is no evidence that there exist retinal ganglion cells with large receptive fields in the human retina that cover the fovea centralis in a redundant manner as in our system. Further, our system does not account for the very fast and simultaneous movement of human eyes, called saccades. Saccades help to scan a broader part of the visual field with the fovea and integrate this information into a detailed map (Purves, 2012). Human eye movement is also controlled by the visual grasp reflex that directs the eyes toward salient events in the periphery of the visual field (Monsell and Driver, 2000). These mechanisms for eye movement could be implemented in the spike encoding algorithm by changing the coordinates for the pooling of DVS pixels for each time step, and thereby virtually moving the center of the visual field. However, this would require additional features to save the movement and integrate it into the SNN.

## Conclusion

This paper presents a new methodology for dynamic visual recognition, inspired by different features of the human visual system. The proposed system is designed to take data from a DVS silicon retina and encodes them into spike trains using an algorithm that mimics the organization and function of retinal ganglion cells. The spike trains are then fed into the brain-like SNN NeuCube, following the retinotopic mapping of photoreceptors from the retina into their neural representations in the primary visual cortex. Two stages of learning, unsupervised and supervised, are performed by NeuCube to extract spatio-temporal patterns from the data and perform a classification task. Results on the benchmark MNIST-DVS dataset have shown that the system can keep up with the classification performance of other methods for dynamic visual recognition. Furthermore, it is possible to dynamically visualize and analyze the activity inside the SNN for a better understanding of the data and the process of their deep learning in the model.

Due to the promising benchmark results and the benefit of the visualization tools for an in-depth understanding of the data and the network processes, we endorse further research on the system. In particular, we suggest the exploration of new learning methods inside NeuCube and of different algorithms for the encoding of DVS data into spike trains.

To date, the highest classification accuracy on the MNIST-DVS dataset has been achieved by Stromatias et al. (2017), who used a spiking convolutional neural network to create a new frame-based dataset, which captures the dynamics of the DVS output and serves as input for a fully-connected classifier that uses stochastic gradient descent. The non-spiking classifier is then mapped to a spiking output layer of LIF neurons. As they mention in their paper, the non-spiking classifier and the spiking output layer can be used with any spiking neural network that has already extracted features from the data in an unsupervised manner. We propose to explore how the connectivity or spiking activity of the NeuCube after the unsupervised learning stage could be used to create a similar frame-based dataset, and how the classifier used by Stromatias et al. (2017) would perform on such a dataset. This way, the biological plausibility of our model could be combined with current state-of-the-art classification algorithms.

We also encourage the development of further benchmark datasets for spike-based visual recognition, e.g., spiking versions of the KTH and the Weizmann datasets of human actions (Laptev and Caputo, 2005; Gorelick et al., 2007). Since the NeuCube architecture is not bound to only consist of neurons representing the visual cortex, future directions can include the integration of our system for visual recognition inside a broader, multimodal methodology, e.g., for the biologically plausible processing of visual and aural data at the same time within the same system. The used DVS format for visual data encoding into spike trains is not a restriction for the proposed SNN method for retinotopic mapping. Learning and other encoding methods for different types of visual data are envisaged to be explored in the future.

## Author contributions

LP the main author, contributes to the spike encoding algorithm, the retinotopic mapping into NeuCube, the choice of MNIST-DVS as a benchmarking dataset, performance evaluation, and paper writing. AW contributes to the initial design of the NeuCube model and partial implementation, and takes part in discussions and reviewing the paper. NK originated the initial idea of this project, and takes part in discussions and reviewing the paper.

### Conflict of interest statement

The authors declare that the research was conducted in the absence of any commercial or financial relationships that could be construed as a potential conflict of interest.

## References

[B1] Berry M. J. Warland D. K. Meister M. (1997). The structure and precision of retinal spike trains. Proc. Natl. Acad. Sci. U.S.A. 94, 5411–5416. 9144251 10.1073/pnas.94.10.5411 PMC24692

[B2] Bichler O. Querlioz D. Thorpe S. J. Bourgoin J. P. Gamrat C. (2012). Extraction of temporally correlated features from dynamic vision sensors with spike-timing-dependent plasticity. Neural Netw. 32, 339–348. 10.1016/j.neunet.2012.02.022 22386501

[B3] Born R. T. Trott A. R. Hartmann T. S. (2015). Cortical magnification plus cortical plasticity equals vision? Vision Res. 111, 161–169. 10.1016/j.visres.2014.10.002 25449335 PMC4400204

[B4] Brader J. M. Senn W. Fusi S. (2007). Learning real-world stimuli in a neural network with spike-driven synaptic dynamics. Neural Comput. 19, 2881–2912. 10.1162/neco.2007.19.11.2881 17883345

[B5] Croner L. J. Kaplan E. (1995). Receptive fields of P and M ganglion cells across the primate retina. Vision Res. 35, 7–24. 7839612 10.1016/0042-6989(94)e0066-t

[B6] Curcio C. A. Sloan K. R. Kalina R. E. Hendrickson A. E. (1990). Human photoreceptor topography. J. Comp. Neurol. 292, 497–523. 10.1002/cne.902920402 2324310

[B7] Delbruck T. (2008). Frame-free dynamic digital vision, in Proceedings of International Symposium on Secure-Life Electronics (Tokyo: Advanced Electronics for Quality Life and Society, University of Tokyo), 21–26.

[B8] Diehl P. U. Cook M. (2015). Unsupervised learning of digit recognition using spike-timing-dependent plasticity. Front. Comput. Neurosci. 9:69. 10.3389/fncom.2015.00099 26941637 PMC4522567

[B9] Gorelick L. Blank M. Shechtman E. (2007). Actions as Space-Time Shapes. Available online at: http://www.wisdom.weizmann.ac.il/~vision/SpaceTimeActions.html (Accessed Aug 29, 2017). 10.1109/TPAMI.2007.70711 17934233

[B10] Henderson J. A. Gibson T. A. Wiles J. (2015). Spike Event Based Learning in Neural Networks. Available online at: http://arxiv.org/pdf/1502.05777.

[B11] Jaygandhi786 (2015). Visual Cortex. Available online at: https://commons.wikimedia.org/wiki/User:OgreBot/Uploads_by_new_users/2015_May_02_15:00 (Accessed Aug 29, 2017).

[B12] Jimenez-Fernandez A. Lujan-Martinez C. Paz-Vicente R. Linares-Barranco A. Jimenez G. Civit A. (2009) From vision sensor to actuators spike based robot control through address-event-representation, in Bio-Inspired Systems: Computational Ambient Intelligence. IWANN 2009. Lecture Notes in Computer Science, Vol. 5517, eds Cabestany J. Sandoval F. Prieto A. Corchado J.M. (Berlin; Heidelberg: Springer).

[B13] Kasabov N. Capecci E. (2015). Spiking neural network methodology for modelling, classification and understanding of EEG spatio-temporal data measuring cognitive processes. Inf. Sci. 294, 565–575. 10.1016/j.ins.2014.06.028

[B14] Kasabov N. Dhoble K. Nuntalid N. Indiveri G. (2013). Dynamic evolving spiking neural networks for on-line spatio- and spectro-temporal pattern recognition. Neural Netw. 41, 188–201. 10.1016/j.neunet.2012.11.014 23340243

[B15] Kasabov N. K. (2014). NeuCube: a spiking neural network architecture for mapping, learning and understanding of spatio-temporal brain data. Neural Netw. 52, 62–76. 10.1016/j.neunet.2014.01.006 24508754

[B16] Kheradpisheh S. R. Ganjtabesh M. Thorpe S. J. Masquelier T. (2017). STDP-Based Spiking Deep Neural Networks for Object Recognition. Available online at: http://arxiv.org/pdf/1611.01421. 10.1016/j.neunet.2017.12.005 29328958

[B17] Krantz J. (2012). Experiencing Sensation and Perception. Upper Saddle River, NJ: Prentice Hall.

[B18] Krieger B. Qiao M. Rousso D. L. Sanes J. R. Meister M. (2017). Four alpha ganglion cell types in mouse retina: function, structure, and molecular signatures. PLoS ONE 12:e0180091. 10.1371/journal.pone.0180091 28753612 PMC5533432

[B19] Laptev I. Caputo B. (2005). Recognition of Human Actions. Available online at: http://www.nada.kth.se/cvap/actions/ (Accessed Aug 29, 2017).

[B20] Lecun Y. Bottou L. Bengio Y. Haffner P. (1998). Gradient-based learning applied to document recognition. Proc. IEEE 86, 2278–2324. 10.1109/5.726791

[B21] Lichtsteiner P. Posch C. Delbruck T. (2008). A 128x128 120 dB 15 μs latency asynchronous temporal contrast vision sensor. IEEE J. Solid State Circ. 43, 566–576. 10.1109/JSSC.2007.914337

[B22] Liu Q. Pineda-García G. Stromatias E. Serrano-Gotarredona T. Furber S. B. (2016). Benchmarking spike-based visual recognition: a dataset and evaluation. Front. Neurosci. 10:496. 10.3389/fnins.2016.00496 27853419 PMC5090001

[B23] Masquelier T. Guyonneau R. Thorpe S. J. (2009). Competitive STDP-based spike pattern learning. Neural Comput. 21, 1259–1276. 10.1162/neco.2008.06-08-804 19718815

[B24] Masquelier T. Thorpe S. J. (2007). Unsupervised learning of visual features through spike timing dependent plasticity. PLoS Comput. Biol. 3:e31. 10.1371/journal.pcbi.0030031 17305422 PMC1797822

[B25] Monsell S. Driver J. (2000). Control of Cognitive Processes: Attention and Performance XVIII. Cambridge, MA; London: MIT Press.

[B26] Mozafari M. Kheradpisheh S. R. Masquelier T. Nowzari-Dalini A. Ganjtabesh M. (2017). First-spike based visual categorization using reward-modulated STDP. Available online at: http://arxiv.org/pdf/1705.09132 10.1109/TNNLS.2018.2826721 29993898

[B27] Nelson R. (1995). Visual responses of ganglion cells, in Webvision: The Organization of the Retina and Visual System, eds Kolb H. Fernandez E. Nelson R. (Salt Lake City, UT: University of Utah Health Sciences Center). 21413404

[B28] Perez-Carrasco J.-A. Serrano C. Acha B. Serrano-Gotarredona T. Linares-Barranco B. (2010). Spike-Based Convolutional Network for Real-Time Processing, in 20th International Conference on Pattern Recognition (ICPR), 2010: 23-26 Aug. 2010, Istanbul, Turkey; proceedings (Piscataway, NJ: IEEE), 3085–3088.

[B29] Perez-Peña F. Morgado-Estevez A. Linares-Barranco A. Jimenez-Fernandez A. Gomez-Rodriguez F. Jimenez-Moreno G.. (2013). Neuro-inspired spike-based motion: from dynamic vision sensor to robot motor open-loop control through spike-VITE. Sensors (Basel) 13, 15805–15832. 10.3390/s131115805 24264330 PMC3871088

[B30] Purves D. (ed.). (2012). Neuroscience. Sunderland, MA: Sinauer.

[B31] Querlioz D. Bichler O. Dollfus P. Gamrat C. (2013). Immunity to device variations in a spiking neural network with memristive nanodevices. IEEE Trans. Nanotechnol. 12, 288–295. 10.1109/TNANO.2013.2250995

[B32] Rosa M. G. P. (2002). Visual maps in the adult primate cerebral cortex: some implications for brain development and evolution. Braz. J. Med. Biol. Res. 35, 1485–1498. 10.1590/S0100-879X2002001200008 12436190

[B33] Serrano-Gotarredona T. Linares-Barranco B. (2015). Poker-DVS and MNIST-DVS. Their history, how they were made, and other details. Front. Neurosci. 9:481. 10.3389/fnins.2015.00481 26733794 PMC4686704

[B34] Stromatias E. Soto M. Serrano-Gotarredona T. Linares-Barranco B. (2017). An event-driven classifier for spiking neural networks fed with synthetic or dynamic vision sensor data. Front. Neurosci. 11:350. 10.3389/fnins.2017.00350 28701911 PMC5487436

[B35] Thorpe S. Gautrais J. (1999). Rank Order Coding, in Computational Neuroscience: Trends in Research, 1998, ed Bower J. M. (Boston, MA: Springer US), 113–118.

[B36] Uzzell V. J. Chichilnisky E. J. (2004). Precision of spike trains in primate retinal ganglion cells. J. Neurophysiol. 92, 780–789. 10.1152/jn.01171.2003 15277596

[B37] van Rossum M. C. Bi G. Q. Turrigiano G. G. (2000). Stable Hebbian learning from spike timing-dependent plasticity. J. Neurosci. 20, 8812–8821. 10.1523/jneurosci.20-23-08812.2000 11102489 PMC6773092

[B38] van Rullen R. Thorpe S. J. (2001). Rate coding versus temporal order coding: what the retinal ganglion cells tell the visual cortex. Neural Comput. 13, 1255–1283. 10.1162/08997660152002852 11387046

[B39] Wei H. Ren Y. (2013). A mathematical model of retinal ganglion cells and its applications in image representation. Neural Process Lett 38, 205–226. 10.1007/s11063-012-9249-6

[B40] Yousefzadeh A. Serrano-Gotarredona T. Linares-Barranco B. (2015). MNIST-DVS and FLASH-MNIST-DVS Databases. Available online at: http://www2.imse-cnm.csic.es/caviar/MNISTDVS.html (Accessed Aug 21, 2017).

[B41] Zhao B. Ding R. Chen S. Linares-Barranco B. Tang H. (2015). Feedforward Categorization on AER Motion Events Using Cortex-Like Features in a Spiking Neural Network. IEEE Trans. Neural Netw. Learn. Syst. 26, 1963–1978. 10.1109/TNNLS.2014.2362542 25347889

